# First Report on the Draft Genome Sequences of *Corynebacterium diphtheriae* Isolates from India

**DOI:** 10.1128/genomeA.01316-16

**Published:** 2016-11-23

**Authors:** Balaji Veeraraghavan, Shalini Anandan, Suresh Kumar Rajamani Sekar, Radha Gopi, Naveen Kumar Devanga Ragupathi, Srilekha Ramesh, Valsan Philip Verghese, Sophy Korulla, Sarah Mathai, Lucky Sangal, Sudhir Joshi

**Affiliations:** a Department of Clinical Microbiology, Christian Medical College, Vellore, Tamil Nadu, India; b Department of Child Health, Christian Medical College, Vellore, Tamil Nadu, India; c World Health Organisation, Country office, New Delhi, India

## Abstract

We report here the draft genome sequences of five *Corynebacterium diphtheriae* isolates of Indian origin. The *C. diphtheriae* isolates TH1141, TH510, TH1526, TH1337, and TH2031 belong to sequence type ST-50, ST-295, ST-377, ST-405, and ST-405, with an average genome size of 2.5 Mbp.

## GENOME ANNOUNCEMENT

*Corynebacterium diphtheriae* is an important human pathogen and the causative agent of the upper respiratory tract infection known as diphtheria (1). The existence of genetic diversity among the strains and its circulation around the world are a serious concern in recent time (2). To the best of our knowledge, this is the first genome report on the *C. diphtheriae* strains isolated from India.

Five *C. diphtheriae* isolates (TH1141, TH510, TH1526, TH1337, and TH2031) were obtained from the patients diagnosed with diphtheria infection at Christian Medical College & Hospital, South India. The presence of *toxA* and *rpoB* genes, typical of *C. diphtheriae*, was confirmed by real-time PCR (RT-PCR) analysis. All the isolates were found to have intermediate resistance to penicillin. Whole-genome sequencing (WGS) was done to elucidate the genetic diversity among the strains. Genomic DNA was extracted with QIAamp DNA minikit (Qiagen, Germany), and the library fragments were prepared using the Ion Plus fragment library kit (Ion Torrent; Life Technologies, USA). Whole-genome sequencing was performed using the Ion Torrent PGM sequencer (Thermo Scientific Fisher Corp., USA) using 400-bp chemistry. The embedded assembler SPAdes tool (version 5.0.0.0) of the Ion Torrent server (version 5.0.4) was used for *de novo* assembly of the next-generation sequencing (NGS) data. Genome annotation was performed using the Pathosystems Resource Integration Centre (PATRIC) (http://www.patricbrc.org) (3) and NCBI Prokaryotic Genome Annotation Pipeline (PGAP) (http://www.ncbi.nlm.nih.gov/genome/annotation_prok/) (4).

The whole-genome assembly of the *C. diphtheriae* isolates (TH1141, TH510, TH1526, TH1337, and TH2031) resulted in the 68, 27, 48, 111, and 136 contigs (≥500 bp) with sequencing coverages of 66×, 96×, 97×, 75×, and 106×, respectively. The number of coding sequences (CDSs) per genome ranged between 2,240 and 2,385, with an average genome size and G+C content of 2.5 Mbp and 53.5%, respectively. The isolates TH1141, TH510, and TH1526 belong to sequence ST-50, ST-295, and ST-377, and TH1337 and TH2031 belong to ST-405, as predicted by the *in-*silico multilocus sequence type analysis (https://cge.cbs.dtu.dk/services/MLST/) (5). Genome annotation by PATRIC predicted a total of 52 tRNAs, seven rRNAs, five antibiotic resistance genes (ARDB), and five virulence genes (VFDB) for the TH1141 genome. The TH510 and TH1526 *C. diphtheriae* genomes harbor 52, seven, five, and four and 53, seven, eight, and seven genes corresponding to the tRNA, rRNA, antibiotic resistance (ARDB), and virulence factor (VFDB), respectively. The TH1337 and TH2031 genomes were found to contain 51, eight, five, and five and 55, seven, five, and five genes related to tRNA, rRNA, antibiotic resistance (ARDB), and virulence factor (VFDB). This study advances our knowledge on the genomic diversity and molecular epidemiology of *C. diphtheriae* in India.

### Accession number(s).

The draft genome sequences of the five *C. diphtheriae* isolates have been deposited in the DDBL/ENA/GenBank database under the accession numbers listed in Table 1.

**TABLE 1:** Genome characteristics and accession numbers of *C. diphtheriae* isolates from India

*C. diphtheriae* isolate	Sequencing coverage (×)	No. of contigs (>500 bp)	Draft genome size (Mbp)	Sequence type	Accession no.
TH1141	66	68	2.46	ST-50	MDYT00000000
TH510	96	27	2.54	ST-295	MDYV00000000
TH1526	97	48	2.45	ST-377	MDYU00000000
TH1337	75	111	2.44	ST-405	MJAQ00000000
TH2031	106	136	2.49	ST-405	MJAR00000000
